# Draft Genome Sequences of Marinobacter Strains Recovered from Utica Shale-Produced Fluids

**DOI:** 10.1128/genomeA.00155-18

**Published:** 2018-04-05

**Authors:** Shantal Tummings, Jenny Panescu, Rebecca A. Daly, Kelly C. Wrighton, Paula J. Mouser

**Affiliations:** a Department of Civil, Environmental, and Geodetic Engineering, The Ohio State University, Columbus, Ohio, USA; b Department of Microbiology, The Ohio State University, Columbus, Ohio, USA

## Abstract

The genomes of three Marinobacter strains, isolated from saline fluids produced from a Utica-Point Pleasant shale well, have been sequenced. These genomes provide novel information on the degradation of petroleum distillates and virulence mechanisms under microaerophilic conditions in fractured shale.

## GENOME ANNOUNCEMENT

Horizontal drilling coupled to hydraulic fracturing well completion methods are the industry standard for recovering hydrocarbon from low-permeability black shales (1, 2). Microorganisms play a role in degrading shale-derived hydrocarbons (3) and altering xenobiotic organic compounds introduced during the fracturing process (4). The bacterial strains Marinobacter persicus UTICA-S1B3, UTICA-S1B6, and UTICA-S1B9 were isolated from saline fluids produced from a Utica-Point Pleasant shale well in Ohio. Cosmopolitan Marinobacter species are distributed across marine lakes, oceans, sediments, and deep mines (5–8), especially where hydrocarbons are present (8, 9), and recently were observed in fractured shale brines (4, 10–12). Here, we describe the genomic sequencing of three Marinobacter strains isolated from fractured shale and highlight their capacity for aromatic compound degradation and bacterial virulence.

The Marinobacter isolates were cultivated from fluids collected on the first day of flowback in Difco marine broth 2216 medium supplemented with 40 mM nitrate at 30°C. Cells were harvested via centrifugation, and genomic DNA was isolated using a DNA minikit (Qiagen, Hilden, Germany), with sequencing performed at the Department of Energy Joint Genome Institute (Walnut Creek, CA, USA). Assemblies were constructed from Illumina MiSeq sequence data (SPAdes version 3.6.2) and generated 94, 96, and 100 contigs for UTICA-S1B3, UTICA-S1B6, and UTICA S1B9, respectively, with a G+C content of 57.7% and 92% genome completeness. Annotation was performed in the Integrated Microbial Genomes platform (Pipeline version 4.12.1) and resulted in 3,287, 3,294, and 3,288 protein-coding genes for UTICA-S1B3, UTICA-S1B6, and UTICA S1B9, respectively. While these strains had an average nucleotide identity (ANI) of 99.9% to each other, their genomes were more distantly related to Marinobacter persicus IRBC-M 10445 (ANI, 83%) and Marinobacter hydrocarbonoclasticus ATCC 49840 (ANI, 78%).

These Marinobacter strains have the genomic potential to degrade toluene and benzene to (methyl)catechol using phenol 2-monooxgenases, further metabolizing catechol through *meta*-cleavage to formate, acrylate, pyruvate, or acetyl-coenzyme A (acetyl-CoA). All three strains contain genes for denitrification and alternative nitrogen source utilization (e.g., urea). Of 35 predicted cytochromes in each genome, 15 cytochromes are annotated for (per)oxidase activity, which may be important for outer membrane processes, including iron oxidation. Unlike other Marinobacter species that utilize a type IV secretion system (13), the Marinobacter strains encode a type VI secretion system that has a known role in the delivery of toxic effectors to other bacteria using a phage-like tubule (14). Specifically, the three strains can target the peptidoglycan of recipient bacterial cells using amidases and proteases or attack outer membranes using phospholipases (14, 15). Amidases may also catalyze the degradation of polyacrylamides (16), which are common additives used during slick-water hydraulic fracturing (17). The isolation of Marinobacter *persicus* UTICA-S1B3, UTICA-S1B6, and UTICA S1B9 provides new insight into hydrocarbon metabolism, polymer degradation, and opportunistic survivability in the shale ecosystem.

### Accession number(s).

The whole-genome sequences for M. persicus UTICA-S1B3, UTICA-S1B6, and UTICA-S1B9 have been deposited in DDBJ/ENA/GenBank under accession numbers PTIV00000000, PTIT00000000, and PTIU00000000, respectively, and can be accessed at the JGI Integrated Microbial Genomes and Microbiome database under the IMG genome identification (ID) numbers 2700989663, 2700989662, and 2700989665, respectively.
